# The Role of Disturbed Mg Homeostasis in Chronic Kidney Disease Comorbidities

**DOI:** 10.3389/fcell.2020.543099

**Published:** 2020-11-12

**Authors:** Cristian Rodelo-Haad, M. Victoria Pendón-Ruiz de Mier, Juan Miguel Díaz-Tocados, Alejandro Martin-Malo, Rafael Santamaria, Juan Rafael Muñoz-Castañeda, Mariano Rodríguez

**Affiliations:** ^1^Maimonides Biomedical Research Institute of Cordoba (IMIBIC), Córdoba, Spain; ^2^University of Córdoba, Córdoba, Spain; ^3^Nephrology Service, Reina Sofia University Hospital, Córdoba, Spain; ^4^Spanish Renal Research Network (REDinREN), Institute of Health Carlos III, Madrid, Spain

**Keywords:** magnesium, chronic kidney disease, hypomagnesemia, cardiovascular disease, mineral metabolism and bone disease

## Abstract

Some of the critical mechanisms that mediate chronic kidney disease (CKD) progression are associated with vascular calcifications, disbalance of mineral metabolism, increased oxidative and metabolic stress, inflammation, coagulation abnormalities, endothelial dysfunction, or accumulation of uremic toxins. Also, it is widely accepted that pathologies with a strong influence in CKD progression are diabetes, hypertension, and cardiovascular disease (CVD). A disbalance in magnesium (Mg) homeostasis, more specifically hypomagnesemia, is associated with the development and progression of the comorbidities mentioned above, and some mechanisms might explain why low serum Mg is associated with negative clinical outcomes such as major adverse cardiovascular and renal events. Furthermore, it is likely that hypomagnesemia causes the release of inflammatory cytokines and C-reactive protein and promotes insulin resistance. Animal models have shown that Mg supplementation reverses vascular calcifications; thus, clinicians have focused on the potential benefits that Mg supplementation may have in humans. Recent evidence suggests that Mg reduces coronary artery calcifications and facilitates peripheral vasodilation. Mg may reduce vascular calcification by direct inhibition of the Wnt/β-catenin signaling pathway. Furthermore, Mg deficiency worsens kidney injury induced by an increased tubular load of phosphate. One important consequence of excessive tubular load of phosphate is the reduction of renal tubule expression of α-Klotho in moderate CKD. Low Mg levels worsen the reduction of Klotho induced by the tubular load of phosphate. Evidence to support clinical translation is yet insufficient, and more clinical studies are required to claim enough evidence for decision-making in daily practice. Meanwhile, it seems reasonable to prevent and treat Mg deficiency. This review aims to summarize the current understanding of Mg homeostasis, the potential mechanisms that may mediate the effect of Mg deficiency on CKD progression, CVD, and mortality.

## Introduction

Magnesium (Mg) is required to maintain cell function, and it has a fundamental role in biological processes such as cell signaling, energy production, metabolism, cell growth and proliferation, synthesis of biomacromolecules, apoptosis, membrane fluidity, and control of cell motility (Romani, 2007). Mg is also required to stabilize RNA and DNA, by the time it facilitates the mechanisms of DNA repair, and it serves as a cofactor for many enzymes (de Baaij et al., 2015). Also, Mg is necessary for appropriate regulation of sodium and potassium transport (Bara et al., 1993).

Over the last decade, the study of Mg disturbances has gained attention given the several molecular processes involving Mg. Both Mg deficiency and hypermagnesemia have been associated with different fatal and non-fatal clinical outcomes.

The kidney plays a key role in Mg handling. Mg deficiency may be observed at any stage of chronic kidney disease (CKD). In the context of CKD, Mg disturbances are associated with an increase in oxidative stress, the production of inflammatory cytokines, sympathetic overactivity, increased adhesion of molecules, inflammation, and the development of cardiovascular disease (CVD). In fact, Mg deficiency has been associated with an increased risk of non-fatal and fatal cardiovascular events. Such association may be supported by the fact that low Mg is related to the development of high blood pressure (BP), renal dysfunction either in native or after kidney transplantation (KT), and vascular calcifications, all of which are determinants of CVD outcomes.

The aim of the present review is to describe the scientific evidence that supports the relationship between Mg and CKD and also the development of different comorbidities associated with CKD. First, a brief description of Mg transport and homeostasis will be presented. Second, an up-to-date description of the current understanding of Mg deficiency and its association with different abnormalities in parathyroid hormone (PTH) secretion, mineral metabolism, bone turnover, and development of CKD will be addressed. Both experimental and human data supporting such an association will be described. Then, the role of Mg in the development of CVD, vascular calcification, and death from cardiovascular causes will be reviewed. Special attention is made to the relationship between Mg disturbances and endothelial dysfunction, coronary artery calcifications (CACs), arrhythmias, and heart failure. Finally, we discuss the limited number of clinical studies on Mg supplementation and future perspectives.

## Mg Transport and Homeostasis

The total amount of Mg in the body is approximately 22.6 g, and the concentration in serum ranges from 2.1 to 3.1 mg/dL. Approximately 1% of the total Mg in the body is located in the extracellular space; 60% is ionized or “free,” 30% is bound to proteins and 10% as phosphate, citrate, or oxalate salts (Kolisek et al., 2019), and bone is an important reservoir of Mg (around 66%) (Green, 1994).

Most Mg is within the intracellular compartment, and the passage to the extracellular space is slow. It is interesting to mention that the Mg concentration in the cytosol and the extracellular space is similar; this is in contrast with other divalent anions such as calcium with an intracellular concentration approximately 20,000-fold lower than in the extracellular compartment.

The Mg gradient between cytosol and cell organelles is almost non-existent (Romani and Scarpa, 2000; Schweigel-Röntgen and Kolisek, 2014). Intracellular Mg is regulated by different types of Mg transporters: transient receptor potential melastatin 7 channel (TRPM7), SLC41A1, SLC41A2, MRS2, TRPM6, and CNNM2 (de Baaij et al., 2015). Although MagT1 was initially described as Mg transporter, most recent evidence indicates that MagT1 gene sequence differs from other known Mg transporter and acts as oxidoreductase subunits of the ER-localized STT3B [catalytic subunit of the oligosaccharyltransferase (OST complex)] (Cherepanova et al., 2014; Shrimal et al., 2015). Different studies have shown the intracellular localization of MagT1 in the endoplasmic reticulum. MagT1 is not expressed in STTB3-B (−/−) HEK293 derived cells, which make unlikely its role as membrane transporter. In addition, the overexpression of MagT1 does not increase intracellular Mg concentration. Therefore, it has been hypothesized that the role of MagT1 in Mg transport is indirect, so STT3-B complex glycosylation mediated by MagT1 is the mechanism through which MagT1 promotes Mg transport activity (Cherepanova and Gilmore, 2016; Cherepanova et al., 2016). Similarly, there is some controversy on whether CNNM2 and CNNM4 act as Mg transporters or exclusively as sensors for other components that finally transport Mg (Kolisek et al., 2019). CNNM2 is highly expressed in the brain, heart, kidney, and liver, and it is of particular interest because its expression is mediated by the amount of Mg contained in the food. Concerning the kidney expression of CNNM2, it is known that variants of human CNNM2 have been implicated in the development of Mg wasting syndrome (dominant hypomagnesemia), which support the role of CNNM2 as Mg transporter (Funato et al., 2017; Kolisek et al., 2019), whereas the deletion of the CNNM2 gene in the brain is associated with disturbed brain development and hypomagnesemia. This information on CNNM2 as Mg transporter has been validated in animal models in which CNNM2 knockout mice present hypomagnesemia and reduced kidney Mg reabsorption (Funato et al., 2017). However, other authors suggest that rather than a transporter itself, CNNM2 acts as a regulator of Mg transport, perhaps through the activation of TRPM7 (Arjona et al., 2014; Sponder et al., 2016). The current understanding of CNNM1 and CNNM3 is scarce, and the specific role is yet to be elucidated.

Homeostasis of Mg is maintained by a controlled balance between the intestinal absorption and renal excretion with a serum concentration ranging from 1.3 to 2.7 mg/dL, although this range may vary between the different laboratories (Pérez González et al., 2009).

Daily intake of Mg is around 300 to 400 mg, and 50% is absorbed in the gastrointestinal tract (ileum and proximal jejunum). Proximal renal tubules reabsorb only 15 to 25% of the filtered Mg; 60–70% is reabsorbed in the ascending limb of the loop of Henle, and the distal tubule reabsorbs a 5 to 10% (Pérez González et al., 2009; de Baaij et al., 2015). Hypomagnesemia is defined as serum Mg concentrations lower than 1.7 mg/dL, whereas hypermagnesemia is considered if serum Mg levels are higher than 2.5 mg/dL.

The primary sources of Mg are seeds, cereals, walnuts, green vegetables, and some types of meat and seafood. There are two processes responsible for intestinal Mg absorption. The first is active transport, which depends on a TRPM6. The second is a passive process that occurs by a paracellular pathway (Pérez González et al., 2009). Renal absorption of Mg occurs in the proximal tubule (15–20%); in the thick ascending loop of Henle, 65 to 70% of Mg is reabsorbed, and a small percentage is reabsorbed in the distal tubule (5–10%) through active transport via the apical TRPM6 pathway. With respect to the active mechanism of Mg transport, TRPM6 channels are located in the basolateral membrane of the cells in the thick ascending limb of Henle loop. However, recent evidence suggests the participation of a sodium-dependent exchange mechanism where Na^+^/K^+^-ATPase pump participates together with low concentrations of Na^+^ (Schlingmann et al., 2002). A large proportion of Mg is reabsorbed in the ascending limb of the loop of Henle through paracellular pathway driven by an electrogenic gradient (positive lumen voltage) generated by the secretion of potassium into the lumen after its reabsorption through the Na-K-2Cl cotransporter.

**FIGURE 1:**
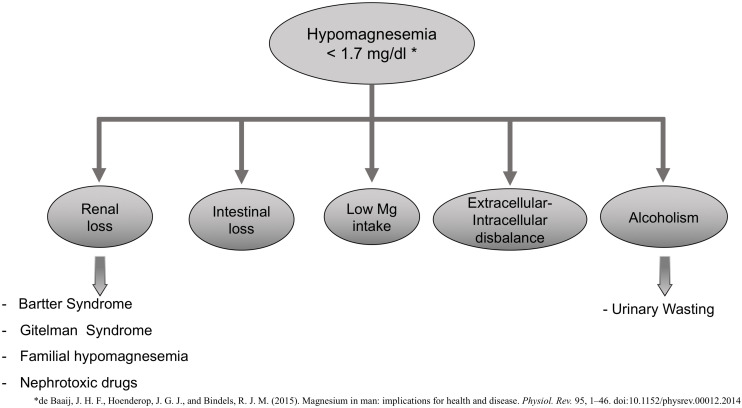
Potential causes of Hypomagnesemia.

Increased renal or gastrointestinal losses may cause hypomagnesemia (plasma Mg concentration <1.3 mg/dL or 0.75 mM) (Figure 1), decreased intake, or a disbalance between extracellular to intracellular Mg (Ahmed and Mohammed, 2019). Bartter and Gitelman syndrome, familial hypomagnesemia with hypercalciuria, and nephrocalcinosis are some of the pathologies associated with an increased renal loss of Mg. There are nephrotoxic drugs [aminoglycoside, amphotericin B, cisplatin, pentamidine, proton-pump inhibitors, calcineurin inhibitors (CNIs) (cyclosporine and tacrolimus), or epidermal growth factor receptor inhibitors] that are known to increase urinary excretion of Mg and thus be the cause of hypomagnesemia (de Baaij et al., 2015).

Hypomagnesemia is also common in alcoholic patients with a prevalence of approximately 30%. These are poorly nourished patients with a large intracellular shift of Mg ions into cells associated with carbohydrate feeding (Crook et al., 2001).

Hypomagnesemia, through several mechanisms, may produce pathophysiological changes responsible for many CKD-associated comorbidities. Low Mg level is frequently observed in CKD, hypertension, cardiac arrhythmias, sudden cardiac death, coronary artery disease, dyslipidemia, or metabolic syndrome (Severino et al., 2019). It has also been described that Mg plays an essential role in the regulation of insulin actions and glucose homeostasis. Hypomagnesemia has been associated with type 2 diabetes mellitus and insulin resistance (Kostov, 2019). More recently, Mg has been linked to energy production. In this regard, Mg binds to ATP to serve as a cofactor during glucose oxidation and modulates some enzymes that participate in the citric acid cycle (Yamanaka et al., 2016). Moreover, Mg enhances mitochondrial isocitrate dehydrogenase, 2-oxoglutarate dehydrogenase, and pyruvate dehydrogenase, all of which participate in energy production. More interestingly, the dysregulation of Mg modifies mitochondrial morphology, so ATP concentration is reduced. This fact is highly relevant because abnormal mitochondrial morphology has been linked to cancer, obesity, type 2 diabetes, and neurodegenerative disorders (Westermann, 2010; Imai and Lu, 2011; Yamanaka et al., 2016).

Low serum Mg levels have also been related to neurological diseases such as migraine, epilepsy, Parkinson disease, or depression (de Baaij et al., 2015).

**FIGURE 2:**
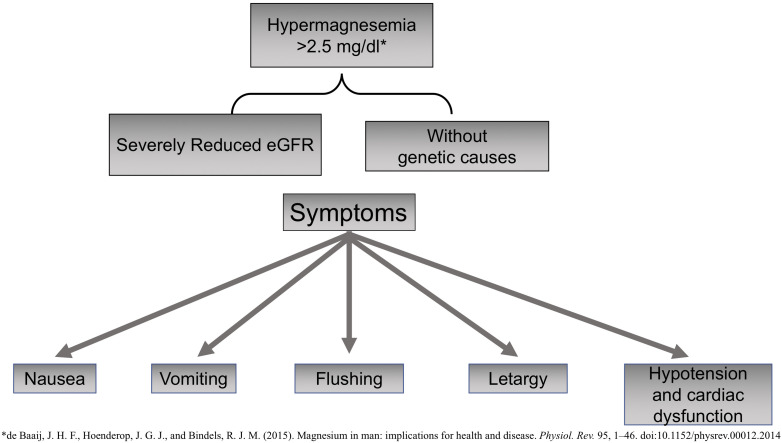
Causes and Symptoms of Hypomagnesemia.

Magnesium also has notable anti-inflammatory properties; therefore, hypomagnesemia is linked to a state of chronic systemic inflammation and oxidative stress (Weglicki, 2012). Mg is an active modulator of the expression and release of proinflammatory neuropeptide substance P, a compound involved in the perception of pain that is promoted by nuclear factor-κB (NF-κB) and the expression of proinflammatory genes and cytokines such as tumor necrosis factor α (TNF-α) (Nielsen, 2018). Hypermagnesemia is not frequent, and so far, no genetic causes have been identified (Figure 2). It may cause nausea, vomiting, flushing, or lethargy. Very high levels of Mg may produce severe cardiac dysfunction with hypotension, asystole, or even death by cardiac arrest.

Hypermagnesemia has been described in claudin 10 knockout mice; claudin 10 is expressed in the thick ascending limb of Henle loop that facilitates paracellular transport of Na^+^. The absence of claudin causes an increase in positive charge (Na^+^) in the lumen, which promotes paracellular Mg transport resulting in hypermagnesemia and nephrocalcinosis (Breiderhoff et al., 2012; de Baaij et al., 2015).

## Hypomagnesemia and CKD

Experimental and human data have been published relative to the association between serum Mg levels, the impairment of kidney function, and renal outcomes in CKD. The abnormal handling of Mg by the kidney may result in either hypomagnesemia or hypermagnesemia (Cunningham et al., 2012; Massy and Drüeke, 2015).

**FIGURE 3:**
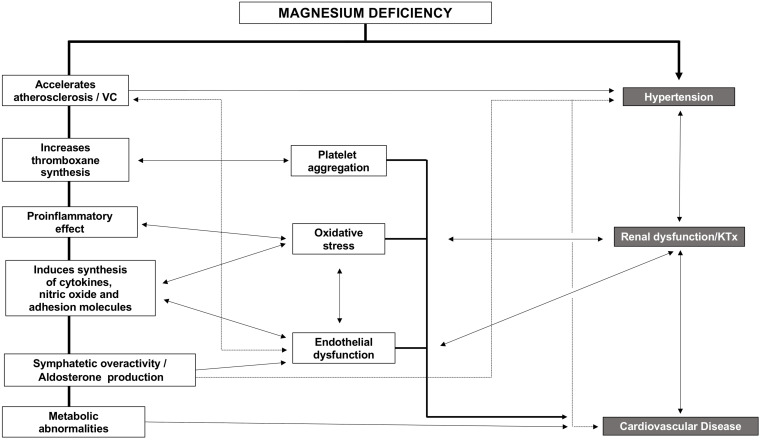
Consequences of Hypomagnesemia.

Strong evidence exists, linking hypomagnesemia and CVD. CVD and CKD share risk factors, so Mg likely impairs kidney function indirectly through the worsening of classic CVD risk factors. Indirect effects may be based on the higher risk of hypertension observed in patients with low Mg (Ascherio et al., 1992). Also, Mg deficiency indirectly impairs kidney function by promoting diabetes and endothelial dysfunction through increasing oxidative stress, inflammation, and the expression of different atherothrombotic factors (Figure 3) that affects different organs and systems such as parathyroid glands, immune system (inflammation), mineral metabolism, bone, kidney, and the cardiovascular system. In the following sections, we will describe the relation between Mg and these systems.

### Mg and the PTH

During CKD, Mg status needs to be taken into consideration to achieve optimal management of mineral metabolism disorders and due to serum Mg has direct effects on PTH regulation and bone.

Cholst et al. (1984) described a case of asymptomatic hypocalcemia in pregnant women after treatment with intravenous Mg sulfate for premature onset of labor. This hypocalcemia was associated with a concomitant increase in plasma Mg and a decrease in PTH levels, bringing up the key role of Mg in the regulation of PTH secretion. Interestingly, plasma calcium remained low despite the normalization of PTH levels after 3 h of Mg infusion.

**FIGURE 4:**
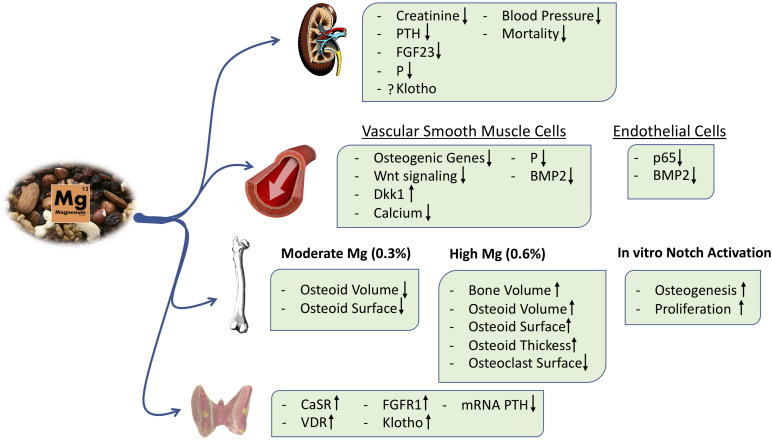
Main effects of Mg Supplementation.

In incubated intact parathyroid glands, an increase in extracellular Mg reduced PTH secretion, and this effect is magnified if PTH is being stimulated by low calcium (Rodríguez-Ortiz et al., 2014; Figure 4). The inhibitory effects of Mg on PTH secretion is mediated through the calcium-sensing receptor (CaSR) (Quinn et al., 2013). Essentially, Mg ions target CaSR in the parathyroid glands, activating the downstream signaling pathway and reducing PTH release (Brown et al., 1987; Zhang C. et al., 2016).

Marked hypomagnesemia also reduces PTH levels even in the presence of hypocalcemia; this phenomenon is referred to as “paradoxical blockade of PTH secretion.” With serum Mg below 1.2 mg/dL, there is a disinhibition of Gα-subunits, which mimics activation of the CaSR and reduction of PTH secretion (Quitterer et al., 2001; Vetter and Lohse, 2002).

In patients undergoing regular hemodialysis, high levels of Mg are associated with low PTH values (Navarro et al., 1999). Sakaguchi et al. (2014, 2018c) showed that, in HD patients, hypomagnesemia was related to high serum PTH, frequent hip fractures, and increased cardiovascular and non-cardiovascular mortality. Very high concentrations of Mg were associated with excessive suppression of PTH. In this respect, plasma Mg concentration appears to be key in the management of CKD-MBD.

### The Effect of Mg on Bone

Around 60% of Mg is located in bone (Gröber et al., 2015). Alfrey and Miller (1973) demonstrated that serum Mg reflects the content of Mg in bone, and the existence of two different pools of Mg in bone was shown, one exchangeable between blood and bone and another that persists in the bone. Additionally, they suggested a role of bone Mg accumulation on renal osteodystrophy. However, there is limited information on the effects of Mg on bone in CKD patients.

Morinière et al. (1988) showed no significant changes in any bone histomorphometric parameter after 8 months of treatment with Mg hydroxide; however, they showed clear tendencies to increase osteoid and decrease osteoclasts resorption surface. In this line of research, Gonella et al. (1988) showed that osteomalacia was prevented by reducing Mg concentration in the dialysis bath. A Mg concentration of >4.5 mg/dL might prevent the *in vitro* formation of hydroxyapatite (Ennever and Vogel, 1981).

In animals, Rude et al. showed that as compared with the control Mg diet (0.063%), an Mg-deficient diet (0.002%) increased plasma calcium concentration and decreased PTH and calcitriol levels. Rats fed a low-Mg diet showed lower trabecular bone volume associated with more osteoclasts and reduced osteoblast activity (Rude, 1998). These findings suggest that, in addition to PTH and vitamin D, Mg is critical for bone remodeling. Other studies by the same group reported similar results in rats fed on diets with sequential Mg depletion, and these bone changes were associated with the presence of a higher amount of proinflammatory and pro-osteoclastogenic cytokines in bone (Rude et al., 2004, 2006, 2009). Thus, Mg supplementation might decrease osteoclast number, being beneficial in a bone disease with high bone resorption, as observed with hyperparathyroidism.

Magnesium promotes passive and active mechanisms that might affect calcium phosphate crystallization. A reduced concentration of Mg ions has a marked effect on nucleation and growth of calcium phosphate crystals. Mg delays the conversion of amorphous calcium precipitates to the more stable apatite phase and promotes the formation of whitlockite, which is a calcium/Mg orthophosphate that may alter bone and soft tissue mineralization. In addition to this passive phenomenon, other studies are showing a direct active role of Mg in the bone that will be exposed later (Ennever and Vogel, 1981; Verberckmoes et al., 2007).

More recently, in a *post hoc* analysis of the CALMAG study, the authors investigated whether there were changes in serum bone turnover markers in a cohort of hemodialysis patients receiving an Mg-based phosphate binder (calcium acetate/Mg carbonate) or the non-calcium-containing phosphate binder, sevelamer. Calcium acetate/Mg carbonate administration increased the serum levels of both calcium and Mg and reduced serum phosphate after 9 weeks of treatment. In this period, Mg-based phosphate binder administration decreased the serum β-crosslap collagen type I C-telopeptides, a marker of bone resorption, and increased the bone-specific alkaline phosphatase. These effects were not observed in the sevelamer group, although both groups showed a similar reduction in PTH levels (Covic et al., 2013). With respect to the active effects of Mg on bone, we have shown that Mg exerts a direct pro-osteogenic effect in an *in vitro* model of mesenchymal stem cells (MSCs) differentiated into osteoblasts, and this effect is at least partially mediated by the activation of the canonical Notch signaling pathway. Furthermore, the Mg transport through the cell membrane via the TRPM7 channel is required to observe Mg effects on osteogenesis, and inhibition of TRPM7 with 2-APB abolished MSC differentiation into osteoblasts (Díaz-Tocados et al., 2017; Figure 4). In addition, in a rat model of renal insufficiency, it was observed that dietary Mg supplementation maintains the number of osteoblasts despite a concomitant reduction in plasma PTH levels; however, excessive amounts of Mg may produce an accumulation of the osteoid in bone (Díaz-Tocados et al., 2017). Extremely high Mg may decrease hydroxyapatite formation in favor of whitlockite (Jang et al., 2015). Thus, Mg supplementation modulates PTH secretion, but it also directly stimulates osteoblast differentiation and maintains osteoclast number in bone; the exact mechanisms are not fully understood, and contemporary studies are needed to clarify the potential effects of Mg on bone and mineral metabolism and the safety of Mg treatment in clinical practice (Muñoz-Castañeda et al., 2018).

A direct effect of Mg on the prevention of renal and CVDs has also been reported in animal models and clinical studies.

### Mg and Kidney Function

Experimental and human data have been published regarding the association between serum Mg levels, the impairment of kidney function, and renal outcomes in CKD. As mentioned, the abnormal handling of Mg by the kidney may result in either hypomagnesemia or hypermagnesemia (Cunningham et al., 2012; Massy and Drüeke, 2015).

#### Experimental Data Related to Hypomagnesemia and Kidney Injury

Since the early 1990s, low-Mg diets were described to induce nephrocalcinosis and impairment of kidney function (Van Camp et al., 1990). Subsequent studies in rats showed proximal renal tubules electron-dense granules in the brush border after only 12 h of Mg-deficient diet. After 7 days, the tubular injury was settled down, as shown by calcium deposits and necrosis of the tubular epithelial (Matsuzaki et al., 2002).

Increased serum phosphate concentration and an excessive tubular load of phosphate induce renal damage through renal tubular injury, interstitial fibrosis, and inflammation (Neves et al., 2004; Sakaguchi et al., 2012, 2018a; Gorostidi et al., 2018; Santamaría et al., 2018). Interestingly, a recent *in vitro* study demonstrated that Mg reduces renal cell toxicity associated with hyperphosphatemia (Sakaguchi et al., 2015; Figure 4). Furthermore, in animal models of CKD, an Mg-deficient diet aggravates tubular damage and down-regulates α-Klotho expression irrespective of serum phosphate levels (Sakaguchi et al., 2018b). It is likely that an Mg-deficient diet results in the up-regulation of mRNA expression of NPT2A cotransporters in proximal tubules, promoting a positive phosphate balance and thus renal tubular injury. Despite the kidney damage and the consequent phosphate overload, a low-Mg diet was associated with low levels of FGF23 (Sakaguchi et al., 2018b), suggesting a deficient phosphate excretion. However, other experiments in animals have shown that dietary Mg supplementation was associated with a decrease in FGF23 levels, perhaps due to a reduction of serum phosphate levels and an increase in renal expression of Klotho (Diaz-Tocados et al., 2017; Figure 4). In Klotho knockout mice, a high Mg intake prevented vascular calcification despite increased serum levels of phosphate and FGF23 (Braake et al., 2019), suggesting other effects of Mg independent of Klotho and phosphate.

Studies in 5/6 nephrectomized rats clearly showed that renal function was better preserved in animals on a diet supplemented with Mg as compared to rats fed a basal Mg diet (0.1%). Different mechanisms could be involved, such as the reduction in intestinal absorption of phosphate mediated by an increase in the dietary intake of Mg, which is also associated with a reduction in vascular calcifications, lower systolic and diastolic BP, and an improvement in endothelial function (Figure 3). However, the beneficial effect of Mg on kidney function seems unlikely to be exclusively due to the reduction of intestinal absorption of phosphate because parenteral administration of Mg was able to reduce vascular calcification without a change in serum phosphate levels (Diaz-Tocados et al., 2017).

#### Clinical Studies Related to Hypomagnesemia and Kidney Injury

The incidence of CKD is increasing worldwide. It would be desirable to identify the factors and mechanisms that may delay CKD progression. In this line, hypomagnesemia has emerged as a factor that should be corrected as a strategy to reduce the progression of CKD. However, most of the information regarding the association between hypomagnesemia and CKD progression is based on observational studies (Joosten et al., 2015; Tin et al., 2015). In a study from the United States, estimated glomerular filtrate rate (eGFR) >60 mL/min/1.73 m^2^ and a low dietary Mg intake (<101 mg/1,000 kcal) were associated with a more rapid decrease in kidney function. This association was maintained irrespective of sociodemographic variables, household income, baseline eGFR, diabetes, and hypertension (Rebholz et al., 2016). In other studies from Iran and Australia, adults with a high intake of Mg had a lower incidence of CKD (Strippoli et al., 2011; Farhadnejad et al., 2016). Diabetic patients with a low Mg intake also showed a more rapid decline in eGFR, together with an increase in proteinuria independently of glycemic control and the use of angiotensin-converting enzyme (ACE) inhibitor/angiotensin receptor blocker (Pham et al., 2005). In a subanalysis of the ARIC study, more than 13,000 African-American patients with eGFR >60 mL/min/1.73 m^2^ were followed up for more than 20 years to prospectively evaluate the incidence of CKD and end-stage of kidney disease (ESKD). Patients with serum Mg less than 1.82 mg/dL showed a two-fold more risk of incident CKD compared to those with serum Mg greater than 2.1 mg/dL irrespective of gender, ethnicity, being diabetic, being hypertensive, and having other comorbidities (Tin et al., 2015). Concerning ESKD, low Mg levels increased by six-fold the risk of ESKD compared to patients with Mg within the normal range. Similarly, another study that included patients with different stages of CKD showed that in those with baseline serum Mg below 1.8 mg/dL, the yearly decline in eGFR was 9.6% higher as compared with 3.5% in subjects with higher values of serum Mg (Van Laecke et al., 2013).

Proteinuria is widely accepted as an independent risk factor for CKD. Proteinuria is also independently associated with hypomagnesemia as it enhances renal Mg wasting (Oka et al., 2018). As such, a combination of proteinuria and hypomagnesemia would aggravate the decline of kidney function.

In critically ill patients with acute kidney injury, there is an association between low Mg and the risk of non-recovery of kidney function (Alves et al., 2013). In this study, the prevalence of hypomagnesemia was significantly higher in patients who did not recover kidney function. These results may be based on the ability of Mg to reduce the negative impact of some medications on the kidney by promoting vasodilation or by reducing inflammation, oxidative stress, and preserving endothelial integrity (Alves et al., 2013). Another possible explanation is the development of endothelial dysfunction that indirectly increases vasoconstriction, BP, and sympathetic nerve overactivity, three well-known risk factors for kidney function impairment (Murasato et al., 1999; Ferrè et al., 2010).

It is important to mention the high prevalence of hypomagnesemia following KT (Nijenhuis et al., 2004). Evidence suggests that the leading cause of hypomagnesemia in this population is the treatment with CNIs as tacrolimus (FK506) and cyclosporine A (CsA). CNIs cause hypercalciuria and hypermagnesuria through the down-regulation of TPRV5, TPRM6, and calbindin-D_28__*k*_ (Nijenhuis et al., 2004). In patients on CsA, hypomagnesemia is associated with a progressive decline of kidney function and a reduction of kidney graft survival. Also, it is suggested that Mg deficiency may exacerbate CsA nephrotoxicity (Holzmacher et al., 2005). In the long term, CNIs also increase the odds of post-transplant diabetes. Consequently, in kidney grafts, the CNI-induced hypomagnesemia and diabetes may contribute to kidney graft function deterioration (Van Laecke et al., 2009). Also, in KT recipients, hypomagnesemia is negatively correlated with pulse wave velocity and thus vascular stiffness, which may contribute to renal function impairment (Van Laecke et al., 2011).

### Mg and CVD

Chronic kidney disease progression is associated with CVD. Actually, a high percentage of mortality in CKD patients is related to cardiovascular causes. Kidney and heart are interconnected organs; dysfunction in one organ may affect the function of the other. This close relationship is defined as cardiorenal syndrome. The mechanism underlying the cardiorenal connection in pathologic conditions remains incompletely understood. Some of the pathophysiological mechanisms whereby kidney and heart are interconnected might be associated with alterations in Mg homeostasis.

Several mechanisms may explain the adverse effects of hypomagnesemia on CVD (Figure 3). Mg deficiency accelerates the atherosclerotic process, increases thromboxane synthesis stimulating platelet aggregation, produces oxidative stress due to its proinflammatory effect, and also induces the synthesis of other cytokines, nitric oxide, and adhesion molecules [vascular cell adhesion molecule 1 (VCAM-1) and intercellular adhesion molecule 1], all of which ends in endothelial dysfunction (Bo and Pisu, 2008). In patients with high cardiovascular risk, low serum Mg is associated with carotid atherosclerosis, independently of other traditional cardiovascular risk factors (Rodríguez-Ortiz et al., 2019).

Additionally, low Mg is associated with metabolic abnormalities such as insulin resistance, hyperglycemia, or dyslipidemia. Mg regulates cellular glucose metabolism and intervenes in phospholipid metabolism and mitochondrial oxidation of long-chain fatty acids (Severino et al., 2019). Serum Mg concentrations ranging from 1.73 to 2.16 mg/dL are associated with a linear decrease in the risk of cardiovascular events. At the same time, Mg intake has a non-linear inverse association with cardiovascular events (Qu et al., 2013).

Pathophysiological processes such as vascular calcifications, hypertension, arrhythmias, heart failure, endothelial dysfunction, and even cardiovascular death are possibly related to abnormal Mg homeostasis. In the following sections, the role of Mg deficiency on cardiovascular disorders is discussed.

#### Endothelial Dysfunction

Magnesium potentiates the production of local vasodilator mediators (prostacyclin and nitric oxide) and facilitates the vascular response to vasoactive substances (endothelin-1, angiotensin II, and catecholamines). The levels of antioxidants (vitamin C, vitamin E, selenium, glutathione peroxidase, superoxide dismutase, and catalase) are also decreased in the context of Mg deficiency (Kostov and Halacheva, 2018). It has been shown that hypomagnesemia decreases endothelial cell proliferation, stimulates the adhesion of monocytes, and potentiates the development of endothelial dysfunction via activation of NF-κB (Kostov and Halacheva, 2018). This correlates with a marked down-regulation of the levels of CDC25B, a member of the CDC25 family of phosphatases that controls the transition from G2 to the M phase of the cell cycle. These modifications are not permanent and are the result of Mg deprivation.

Low Mg causes endothelial dysfunction by generating a proinflammatory, prothrombotic, and proatherogenic environment (Maier et al., 2004b). Maier et al. (2004a) demonstrated a direct effect of Mg in maintaining endothelial function due to its protective effect against atherosclerosis and its role in promoting the growth of collateral vessels in chronic ischemia. High Mg might facilitate the re-endothelialization of damaged vessels.

In the vascular wall, Mg regulates collagen and elastin turnover, as well as matrix metalloproteinase activity; it also reduces calcium deposition in the elastic fibers, so the elasticity of the vessels is maintained. It has been described that hypomagnesemia may increase BP through several mechanisms: vasoconstriction, endothelial dysfunction, low-grade vascular inflammation, atherosclerosis, vascular remodeling, vascular aging, low-stress tolerance, and sodium retention. These changes result in arterial stiffness and hypertension (Kostov and Halacheva, 2018). We have reported that recombinant TNF-α increases the levels of mRNA and protein of BMP2 and p65 in human umbilical vein endothelial cells (HUVECs); the addition of MgCl_2_ at 1.4 and 2.6 mM reduced significantly the levels of both proteins providing an additional mechanism by which Mg may protect against endothelial dysfunction (Diaz-Tocados et al., 2017). Similarly, in HUVECs incubated with different concentrations of Mg (low: 0.1 mM, control: 1 mM, high: 5 mM), the inflammatory response to LPS was increased in cells cultured in low Mg and suppressed in high Mg. This effect is mediated through the toll-like receptor 4/NF-κB pathway (Almousa et al., 2018).

#### Mg and Vascular Calcification

A wide number of factors are responsible for the development of arterial calcifications in CKD. In addition to high calcium, phosphate, the Ca-P product, uremic toxins, and other abnormalities, low Mg is also involved in the development of vascular calcifications. Indeed, in a group of patients receiving peritoneal dialysis followed up for more than 3 years, the serum Mg concentration discriminated patients with vascular calcification irrespective of the Ca-P product (Meema et al., 1987).

There are many cases of hyperphosphatemia and Mg deficiency. The administration of phosphate binders containing Mg reduces serum phosphate levels and improves hypomagnesemia, and they may help to reduce vascular calcifications. In experimental animals, dietary Mg supplementation improves phosphate handling and prevents calcifications (Diaz-Tocados et al., 2017).

Low serum Mg level is also associated with CAC, even in a population without previous evidence of CVD (Lee et al., 2015; Posadas-Sánchez et al., 2016; Rosique-Esteban et al., 2018). In CKD G4 patients, Mg supplementation reduces CAC progression (Sakaguchi et al., 2019). In stage G3-4 CKD patients, Mg could be an efficient strategy to improve outcomes given the demonstrated effect of Mg oxide (MgO) supplementation in the reduction of CAC progression. In this population, MgO reduced the percent change in CAC scores as compared with controls. However, no benefit was observed on thoracic aorta calcification, which warrants further investigation (Sakaguchi et al., 2019).

Experimental studies also observed a beneficial effect of Mg supplementation on vascular calcification. We have shown *in vitro* that Mg supplementation prevented phosphate-induced calcification in VSMC; this is mediated by the inhibition of a potent osteogenic cell signaling, the Wnt/β-catenin pathway (Montes de Oca et al., 2014). This effect is independent of hydroxyapatite formation because it is prevented by the blockade of cellular transport of Mg (Montezano et al., 2010; Kircelli et al., 2012; Louvet et al., 2013; Montes de Oca et al., 2014). *In vivo*, we observed that dietary Mg supplementation prevents and even reverse vascular calcification in an experimental model of 5/6 nephrectomy (Diaz-Tocados et al., 2017; Figure 4).

#### Mg and Hypertension

Experimental, clinical, and epidemiological studies have shown an inverse relationship between Mg and BP level, suggesting a relevant role of hypomagnesemia in the pathogenesis of hypertension (Touyz et al., 2002), but the mechanisms are not clear. Hypertensive patients with high renin activity have significantly lower serum Mg levels than normotensive subjects, and plasma renin activity is inversely related to the serum Mg (Resnick et al., 1983). Hypertensive patients with uncontrolled BP might have hypomagnesemia (Cunha et al., 2012).

Another meta-analysis supports the evidence of an inverse dose–response relationship between dietary Mg intake and the risk of hypertension. However, the evidence about the relationship between serum Mg concentration and hypertension is limited (Han et al., 2017).

The antihypertensive effects of Mg supplementation have been widely suggested. In a meta-analysis, Zhang X. et al. (2016) showed that Mg supplementation at a median dose of 368 mg/d for a median duration of 3 months significantly reduced systolic BP by 2.0 mmHg (95% confidence interval, 0.43–3.58) and diastolic BP by 1.78 mmHg (95% confidence interval, 0.73–2.82); these reductions were accompanied by an increase in serum Mg of 0.12 mg/dL (95% confidence interval, 0.07, 0.17 mg/dL) as compared with placebo (Zhang X. et al., 2016).

As previously mentioned, Mg may modulate BP through different mechanisms (Teo and Yusuf, 1993). In a spontaneously hypertensive animal model, it has been observed that Mg supplementation reduced the increase in BP induced by CsA (Pere et al., 2000). More importantly, Mg deficiency aggravated renal CsA-induced nephrotoxicity, whereas Mg supplementation prevented renal morphological changes and reduced proteinuria by 65% (Mervaala et al., 1997; Pere et al., 2000).

The exact mechanisms mediating the antihypertensive effect of Mg supplementation are not entirely known. Mg transport into the vascular smooth muscle cells may be the main determinant to reduce the contraction capacity of the VSMC. Increased concentrations of extracellular Mg promote vasodilation and decrease agonist-induced vasoconstriction. By contrast, low Mg concentrations cause VSMC contraction and enhance agonist-induced vasoconstriction (Touyz, 2003). Mg may also modify vascular tone through changes in Na^+^/K^+^ ATPase activity, which control Na^+^ and K^+^ transport and the levels of the vasodilators cyclic AMP and GMP (Touyz et al., 1991). Mg also decreases endothelin-1 and enhances vasodilation by augmenting nitric oxide (de Baaij et al., 2015). Additionally, animal studies have shown abnormalities in aldosterone regulation associated with hypomagnesemia. Hypomagnesemia promotes the up-regulation of aldosterone, which in turn worsens Mg deficiency through the down-regulation of TRPM7 transporters resulting in high BP. On the contrary, the supplementation of Mg decreases serum aldosterone levels (Sontia et al., 2008).

#### Arrhythmias

The antiarrhythmic effects of Mg have been well documented; this effect of Mg is mediated by changes in the activity of sodium, calcium, and potassium ionic channels with significant consequences on the duration of the action potential, cell excitability, and contractility (Agus and Agus, 2001). Numerous studies have evaluated the role of Mg in maintaining cardiac rhythm and preventing arrhythmias. An association between low serum Mg levels and the development of atrial fibrillation has been described (Khan et al., 2013). In a systematic review and meta-analysis, the post-operative administration of Mg appears to reduce the risk of atrial fibrillation after cardiothoracic surgery (Fairley et al., 2017), and it has been recently reported that the use of intraoperative Mg is associated with a reduction in post-operative arrhythmias (Mohammadzadeh et al., 2018; Salaminia et al., 2018). A meta-analysis suggests that Mg sulfate can be used safely and effectively in the prevention of arrhythmias (Salaminia et al., 2018). In another clinical trial, Mg administration did not significantly improve the incidence of arrhythmias in patients with hypomagnesemia or normomagnesemia after coronary artery bypass grafting. There was no significant correlation between post-operative serum levels of Mg and arrhythmia (Carpenter et al., 2018). Nevertheless, more studies are needed to demonstrate the effectiveness of Mg administration, dose, and length of treatment, in preventing arrhythmias.

The mechanisms whereby Mg intervenes in arrhythmias are not well defined yet. During hypomagnesemia, tachyarrhythmias, prolonged repolarization time, and electrocardiographic disturbances are frequently observed (Topf and Murray, 2003). *In vitro*, data demonstrate that Mg is involved in the generation and maintenance of resting membrane potential and regulating action potential through its effect on multiple ion channels (Leenders and Vervloet, 2019). Hypomagnesemia also predisposes to hypokalemia, enhancing the possibility of arrhythmias (Dinicolantonio et al., 2018).

#### Mg, Heart Failure, and Cardiovascular Death

Hypomagnesemia produces a decrease in cardiac contractility and increases the vascular tone. The administration of furosemide and digoxin is often used to treat heart failure and increase the renal excretion of Mg with a worsening of hypomagnesemia (Kunutsor et al., 2016; Dinicolantonio et al., 2018). Furthermore, low serum Mg is independently associated with the risk of developing heart failure (Kunutsor et al., 2016). However, baseline Mg level does not predict a worse outcome in patients with heart failure (Vaduganathan et al., 2013).

In the general population, it has been observed a protective effect of Mg against CVD (Del Gobbo et al., 2013). Lower serum Mg levels were associated with an increased risk of cardiovascular death (Reffelmann et al., 2011; Kieboom et al., 2016; Zhang et al., 2018). In a meta-analysis performed in elderly patients with CKD and reduced left ventricular systolic function, it was observed that high serum Mg levels (>2.55 mg/dL) were associated with an increased risk of cardiovascular death (Angkananard et al., 2016). However, Vaduganathan et al. (2013) have shown that the excess risk of cardiovascular death associated with higher Mg levels is likely attributable to older age, renal dysfunction, and worse baseline New York Heart Association functional class rather than Mg levels *per se*.

In CKD and ESKD patients, the relationship between hypomagnesemia and CVD and mortality has been increasingly reported in observational studies, although as renal function deteriorates, the renal Mg excretion decreases, resulting in higher serum Mg levels (Ikee, 2018). There is an independent inverse association between serum Mg and all-cause mortality in European hemodialysis patients (de Roij van Zuijdewijn et al., 2015). Likewise, Lacson et al. (2015) found that, in American hemodialysis patients, elevated serum Mg levels (>2.5 mg/dL) were associated with better survival than low serum Mg levels (<1.56 mg/dL). In Japanese non-diabetic hemodialysis patients, serum Mg levels higher than 2.5 mg/dL were associated with improved survival and a 62% reduction of mortality risk. Kaplan–Meier analysis showed a significantly higher incidence of death in patients with serum Mg levels lower than 2.5 mg/dL (Shimohata et al., 2019). Furthermore, in a prospective study in Japanese hemodialysis patients, hypomagnesemia showed significantly worse 3-year cumulative survival for all-cause mortality; however, hypomagnesemia was not an independent risk factor for mortality, and it was associated with malnutrition (Mizuiri et al., 2019). In recent systematic review and meta-analysis of 20 publications, there was a strong association between hypomagnesemia and the risk of all-cause mortality in patients with CKD and ESKD (Xiong et al., 2019).

Finally, it has been documented that some beneficial effects of Mg may be achieved through the protection of organs against the injury induced by inflammation.

## The Effects of Mg Supplementation, Current Evidence

The protective effect of dietary Mg on CVD is based on the inverse association between Mg intake and insulin resistance, hyperglycemia, dyslipidemia, hypertension, and decreased markers of inflammation. A meta-analysis, including two independent American and Japanese cohort studies, has shown a strong inverse association between Mg intake and heart failure (Fang et al., 2016b). Sakaguchi et al. (2019) have recently published that Mg supplementation prevents CAC progression in CKD patients.

Supplements of Mg protect against the hemodynamic instability associated with the electrolyte abnormalities observed in heart failure where treatments with furosemide, digitalis, and ACE inhibitors could favor Mg excretion (Carpenter et al., 2018). Nevertheless, reports on the associations between dietary intake or supplements of Mg and arrhythmias are scarce. A low Mg intake may result in Mg depletion and arrhythmias (atrial fibrillation and flutter) in postmenopausal women and that Mg supplementation reverses these arrhythmias (Nielsen et al., 2007). However, a more recent prospective study, including American participants, has not shown a significant relationship between Mg intake and the incidence of atrial fibrillation (Misialek et al., 2013). Nevertheless, some authors recommend that hypomagnesemia should be corrected by oral Mg supplementation to prevent cardiac rhythm disorders (Carpenter et al., 2018).

Maintenance of optimal Mg status may also be an appropriate strategy to help control the BP (Kostov and Halacheva, 2018). A negative correlation between dietary Mg intake and BP has been observed, and supplementary oral Mg intake significantly reduced systolic and diastolic BP (Cunha et al., 2017; Carpenter et al., 2018) and ameliorated subclinical atherosclerosis (Cunha et al., 2017). Concerning cardiovascular death, in a meta-analysis, which included 400,000 adults, the dietary Mg intake was inversely associated with the risk of coronary heart disease, heart failure, and sudden cardiac death (Fang et al., 2016a). Repletion of Mg has been found to reduce the risk of arrhythmias and death after acute myocardial infarction, and it may also reduce sudden cardiac death (Dinicolantonio et al., 2018). Moreover, in a meta-analysis evaluating dose–response of prospective cohort studies, increasing dietary Mg intake was associated with a reduced risk of heart failure and all-cause mortality (Fang et al., 2016b). In a Mediterranean population with high cardiovascular risk, a randomized clinical trial showed that dietary Mg intake was inversely related to the mortality risk. However, no significant association was observed between Mg intake and major cardiovascular events (Guasch-Ferré et al., 2014).

Supplementation of Mg may be a therapeutic strategy for the management of CVD (Severino et al., 2019). The majority of recent studies show an inverse correlation between dietary Mg intake and serum Mg levels with the risk of CVD and mortality (Muñoz-Castañeda et al., 2018). High Mg intake is associated with a lower risk of major cardiovascular risk factors (mainly metabolic syndrome, diabetes, and hypertension) and less incidence of stroke and CVD (Rosique-Esteban et al., 2018). Mg supplementation should be considered in patients with a high risk of Mg deficiency and even more if they have high cardiovascular risk.

Several randomized, double-blind, placebo-controlled trial has also shown an improvement in endothelial function after oral Mg supplementation using different doses and duration of treatment. Mg supplementation has been used successfully in patients with coronary artery disease (Shechter et al., 2000), in hypertensive women (Cunha et al., 2017), and diabetic elderly patients (Barbagallo et al., 2010). A systematic review and meta-analysis showed that the use of oral Mg might improve flow-mediated dilation (Darooghegi Mofrad et al., 2018). However, Mg supplements did not improve endothelial function and cardiovascular risk markers in overweight and obese middle-aged and elderly adults (Joris et al., 2017). Supplementation of choline plus Mg oxide was more effective in improving inflammation and endothelial dysfunction in diabetic patients than supplementation with choline or Mg alone (Rashvand et al., 2019). Also, with respect to inflammation, in a large cohort of postmenopausal women, dietary Mg intake was inversely associated with the values of C-reactive protein (CRP), interleukin 6 (IL-6), TNF-α, VCAM-1, and E-selectin (Chacko et al., 2010).

One main effect of Mg supplementation is obtained through the decrease in inflammation. The protection of Mg against cardiovascular and renal inflammatory actions of aldosterone was demonstrated by Sontia et al. (2008). Treatment with recombinant aldosterone plus 0.9% of NaCl in drinking water promoted renal and cardiovascular expression of IL-6, VCAM-1, and COX2 in C57B6 mice. Aldosterone overload also decreased renal expression of the TRPM7 channel. Dietary Mg (0.75%) attenuated renal and cardiac IL-6 content and decreased renal VCAM-1 and cardiac COX2 expression and increased renal expression of TRPM7.

Chronic systemic inflammation and oxidative stress are also common features of CKD and are associated with alterations in mineral metabolism (Stenvinkel et al., 2005). In uremia, there is an increase in the production of reactive oxygen species and cytokines, which favor calcification in CKD rats (Agharazii et al., 2015). Oxidative stress may be linked to hypomagnesemia, even at the very early stages of CKD (Figure 3).

*In vitro*, low Mg promotes the up-regulation of NF-κB (Van Laecke et al., 2012) and also increases the levels of IL-1β and TNF-α (Massy and Drüeke, 2015). *In vivo*, Mg deficiency mediates leukocyte activation and the release of several proinflammatory cytokines (Tejero-Taldo et al., 2006). In humans, hypomagnesemia is also associated with an elevation in CRP and worsens insulin resistance in diabetic patients (Kim et al., 2010).

## Conclusion and Final Remarks

To date, the decision-making of prescribing Mg supplements to patients with Mg deficiency remains uncertain. Still, there is no evidence strong enough to make a formal recommendation to prescribe Mg supplements. However, there are data available that strongly suggest the benefits of Mg on vascular calcification, CVD, and kidney function. Thus, it appears that Mg supplementation, if indicated, may slow down the development of such negative morbidities.

From this ample bibliography related to Mg, one idea prevails; it is necessary to control the levels of Mg so that low levels of serum Mg should not be left untreated. The deleterious effects of Mg deficiency have been sufficiently demonstrated. However, it is also mandatory to learn what patients will benefit most from Mg supplementation and what the best protocol is to this end. It is likely that, in elderly patients with ESKD, Mg supplementation should be done carefully to avoid Mg accumulation due to the reduced renal function; for this reason, more clinical and experimental studies are necessary to learn when and how we must manage Mg to treat hypomagnesemia.

## Author Contributions

CR-H, MVP-R, JMD-T, AM-M, RS, JRM-C, and MR wrote the article. All authors contributed to the article and approved the submitted version.

## Conflict of Interest

The authors declare that the research was conducted in the absence of any commercial or financial relationships that could be construed as a potential conflict of interest.
